# Burden of anxiety, depression and stress among older adults living in South-East Asia: Protocol for a systematic review and meta-analysis

**DOI:** 10.1136/bmjopen-2025-106812

**Published:** 2025-11-26

**Authors:** Md Saimul Islam, Jeetesh V Patel, Helal Uddin Ahmed, Aliya Naheed, Paramjit Gill

**Affiliations:** 1 Warwick Centre for Global Health, University of Warwick, Coventry, UK; 2 Non-Communicable Diseases, Nutrition Research Division, icddr,b, Dhaka, Bangladesh; 3 Child Adolescent and Family Psychiatry, Faridpur Medical College, Faridpur, Bangladesh

**Keywords:** EPIDEMIOLOGY, MENTAL HEALTH, Aging, Depression & mood disorders, Anxiety disorders, Stress, Physiological

## Abstract

**Abstract:**

**Introduction:**

Depression, anxiety and stress are major contributors to the global burden of diseases. The ageing population faces an escalating burden of these conditions, and half of the cases are largely undiagnosed. Yet a paucity of epidemiological data limits understanding the full scope of the disease burden among older adults. This protocol outlines a systematic review to estimate the prevalence and incidence of anxiety, depression and stress among older people (60 years and above) and to identify contributing factors across South-East Asian countries.

**Method and analysis:**

A study protocol for a systematic review and meta-analysis has been registered in PROSPERO. The research team will systematically search, appraise and synthesise observational studies following the Preferred Reporting Items for Systematic Reviews and Meta-Analyses (PRISMA) guidelines. Comprehensive searches will be conducted from inception to May 2025 across PubMed (NCBI), MEDLINE (Ovid), Web of Science, Cochrane Library, Scopus (Elsevier) and PsycINFO (APA), supplemented by grey literature from government reports, the WHO Library and Google Scholar. Two investigators will independently screen titles and abstracts, review full-text articles published in the English language and extract data, with discrepancies resolved by a third reviewer. Methodological quality and risk of bias of the included studies will be assessed using standardised tools. Primary outcomes are the prevalence and incidence of depression, anxiety and stress. Secondary outcomes include variations in the prevalence and incidence of these conditions based on sociodemographic factors, as well as associated risk factors that differ across regional contexts. Data will be pooled via meta-analysis where feasible or narratively synthesised if heterogeneity precludes quantitative synthesis. The systematic review will provide a comprehensive understanding of the burden of anxiety, depression and stress among older people in South-East Asia. This novel evidence will guide policymakers and healthcare practitioners in developing targeted interventions and generating essential evidence for supporting policy development in the region.

**Ethics and dissemination:**

Ethical approval will not be required as this study will not involve collection of original data. The findings will be disseminated through publications in a peer-reviewed journal and presentations at scientific conferences.

**PROSPERO registration number:**

CRD42024609033.

**STRENGTHS AND LIMITATIONS OF THIS STUDY**

- This systematic review is the first attempt in South-East Asia to estimate the prevalence and incidence of depression, anxiety and stress among older adults.
- Joanna Briggs Institute (JBI) tool and Grading of Recommendations Assessment, Development and Evaluation (GRADE) approach will be adopted to assess the quality of the studies.
- Multiple electronic databases and grey literature sources will be searched to maximise inclusion of relevant studies and minimise the publication bias.
- Articles published in English will be reviewed, which may exclude relevant research in local languages and introduce language bias.
- Included studies may lead to substantial heterogeneity due to different study designs and may limit the conduct of a meta-analysis.

## Introduction

Older adults experience a high burden of depression, anxiety and stress, which are major determinants of disability in later life.^1^,^3^ These mental health disorders remain undiagnosed and untreated in many developing countries. In 2023, the global population adults aged 60 years and above reached 1.1 billion, and approximately 14% affected by anxiety and depression.^4 5^ A global review indicates that more than one in seven older adults have experienced either anxiety-, depression- or stress-related conditions.^6^ These conditions are collectively contributing to 10.6% of years lived with disability (YLD) among older adults.^5 6^ Nearly two-thirds of older people are living in Asia, where the burden of mental health disorders is disproportionately concentrated across several countries of Asia.^7 8^

As of 2024, one in every four individuals globally is living with a mental disorder (MD).^9^ The burden of MDs is substantially higher in the WHO South-East Asia Region (SEARO) compared with other regions, such as Africa and Western Pacific.^10 11^ Rapid population ageing in the SEARO which includes Bangladesh, Bhutan, the Democratic People’s Republic of Korea, India, Maldives, Myanmar, Nepal, Sri Lanka, Thailand and Timor-Leste has heightened public-health concerns related to mental disorders. Specifically, anxiety and depression have emerged as the most prevalent mental disorders, contributing to over one-sixth of years lived with disability (YLDs) among older adults.^12^ Stress is also a common concern in later life and emerging evidence suggests that its prevalence is rising and may nearly double over the next decade.^13^ However, many common mental health issues remain undiagnosed in many older adults due to cultural stigmas and limited mental health services.^14 15^

Regional and country level estimates of mental disorders are available from limited sources, notably the Global Burden of Disease and WHO report.^8 16^ Within the WHO-SEARO, empirical evidence on common mental disorders among older adults is sparse and fragmented.^17^ Existing estimates are often derived using indirect methods (eg, mathematical modelling), providing a broader estimate, which does not necessarily represent variability of the estimates within a country. Yet such estimates are crucial for understanding the epidemiology of anxiety, depression and stress, and informing the development of locally appropriate mental health policies and services.^17 18^ Previous reviews either aggregate data across the whole of Asia or restrict analysis to South Asia,^6 19^ and only a small number of studies on mental disorders in older adults have been included in few systematic reviews.^20^,^25^ These studies are unevenly distributed across countries and methodologically heterogeneous. Consequently, the current evidence base is insufficient to estimate precise prevalence or incidence of anxiety, depression and stress for older adults either at regional or country level across WHO-SEARO countries.

This protocol outlines a systematic review to estimate the prevalence and incidence of anxiety, depression and stress among older adults in the WHO-defined South-East Asian region. In addition, the review will explore variations in these conditions across key characteristics of the participants.

### Review questions

1. What are the prevalence and incidence rates of anxiety, depression and stress among the older adults in the WHO South-East Asia region?
2. How do the prevalence and incidence of anxiety, depression and stress among older adults vary according to demographic, social and clinical characteristics?

## Methodology

### Design and reporting

This protocol is registered with the PROSPERO International Prospective Register of Systematic Reviews (registration number: CRD42024609033) and reported with Preferred Reporting Items for Systematic Reviews and Meta-Analyses Protocols (PRISMA-P). The review strategy was developed based on the PECOS framework (Population, Exposure, Comparator, Outcomes and Settings). Eligibility criteria were defined as follows: population: individuals aged 60 years and above from countries in the WHO SEARO, including community-dwelling older adults and those attending outpatient clinics or admitted to hospitals, with or without other health conditions; exposure: presence of depression, anxiety, stress; comparator: not applicable for prevalence estimates. For incidence analyses (longitudinal/cohort designs), the comparator group will comprise participants without depression, anxiety or stress at baseline; outcome: prevalence or incidence of depression, anxiety and stress among the older adults. The primary outcome of the study is the pooled prevalence and pooled incidence estimate of depression, anxiety and stress among in older adults in the WHO-SEARO. Secondary outcomes includes assessing variations in prevalence or incidence across countries and to identify sociodemographic characteristics and clinical factors related to depression, anxiety and stress in older adults; settings: WHO-SEARO countries as follows Bangladesh, Bhutan, Democratic People’s Republic of Korea, India, Maldives, Myanmar, Nepal, Sri Lanka, Thailand and Timor-Leste. The PRISMA-P was used for the reporting of protocol (online supplemental file 1).

### Eligibility criteria

We will include peer-reviewed primary observational studies including longitudinal cohort, cross-sectional and case–control studies that report outcomes related to depression, anxiety and stress in older adults. We will screen existing systematic reviews of prevalence or incidence only to identify additional eligible primary studies and to contextualise our findings; these reviews will be documented as background sources and not included in the evidence synthesis. We will include studies reporting prevalence or incidence of depression, anxiety and stress among older adults (≥60 years) residing in countries within the WHO SEARO.^26^ The WHO SEARO region comprises Bangladesh, Bhutan, Democratic People’s Republic of Korea, India, Maldives, Myanmar, Nepal, Sri Lanka, Thailand and Timor-Leste. We will include studies that use validated screening tools or diagnostic criteria to assess depression, anxiety and stress. Types of outcome measures: the number of positive cases out of total eligible participants, reported prevalence or incidence estimates of depression, anxiety and stress.

We will exclude studies that have the following criteria: (1) experimental studies, editorials, commentaries and case series; (2) studies focusing on individuals below the age of 60 or including mixed-age populations when data for older adults cannot be distinguished; (3) studies that do not explicitly report outcomes related to depression, anxiety, stress or any combination of these conditions; (4) studies with inaccessible full texts, such as conference abstracts or unpublished data.

### Search strategy

A comprehensive search strategy will be used to identify relevant articles from multiple databases and sources: PubMed (NCBI), MEDLINE (Ovid), Web of Science, Cochrane Library, Scopus (Elsevier) and PsycINFO (APA). Additionally, we will manually search the WHO Global Health Library, national and subnational reports from WHO South-East Asia regional countries and government websites. A combination of MeSH terms and free-text keywords related to mental health, the older population and the geographical region will be used. All databases will be searched from inception to May 2025 and only studies published in English will be included in the review. Online supplemental file 2 presents the primary search strategy to be conducted in PubMed/Medline for searching other databases.

### Study selection and data extraction

All identified studies will be imported into reference management software (eg, Rayyan) for deduplication. Two independent reviewers will screen the titles and abstracts of all studies to assess their relevance based on the inclusion and exclusion criteria. Disagreements will be resolved through consensus following a consultation with a third reviewer. Full texts of the eligible studies will be reviewed if they meet eligibility criteria. When essential information is missing, the corresponding author will be contacted, and efforts will be made to contact other authors if the corresponding author is not reachable after three attempts. A final list of included studies will be compiled, and reasons for exclusion will be documented.

A standardised data extraction form will be used to collect relevant information from the studies deemed eligible following full-text review as follows: (1) study characteristics: authors, publication year, country, study design, sample size and setting; (2) participant characteristics: age, sex, education, socioeconomic status and any physical illness of the older adults; (3) outcome measures: prevalence of depression, anxiety, stress or combined outcomes; (4) methodology: valid assessment tools for depression, anxiety and stress and statistical methods used for reporting univariate, bivariate and multivariable analysis. Extracted data will be entered into a database (eg, Review Manager) for further analysis. When multiple article report similar evidence, we will prioritise the most recently published results.

### Assessment of methodological quality

The methodological quality of the included studies will be assessed using the Joanna Briggs Institute (JBI) Critical Appraisal Tools.^27^ These tools are tailored to specific study designs, such as cohort, case–control and cross-sectional studies, allowing a systematic evaluation of the risk of bias and overall quality. Two independent reviewers will assess each study using the appropriate JBI tool, focusing on key domains such as study design, representative sample size, data collection methods and statistical analysis. If the decision to include a study varies between two reviewers, this issue will be resolved through consultation with a third reviewer.

The quality of evidence across studies will be assessed using the Grading of Recommendations Assessment, Development and Evaluation (GRADE) methodology.^28^ This approach evaluates evidence quality based on factors such as study limitations, inconsistency of results, indirectness, imprecision and publication bias. GRADE will be applied to determine the strength of evidence for the overall findings on depression, anxiety and stress in older adults.^29^ Evidence will be categorised as high, moderate, low or very low quality. The results of the JBI appraisal and GRADE assessment will be documented and used to inform the interpretation of the findings generated from the final analyses. Inclusion of low-quality studies will be considered with caution while reporting the prevalence or incidence, and their impact on conclusions will be discussed.

### Potential confounding variables

We will extract potential confounders at the individual level (age bands within older adults; sex; socioeconomic status; marital/living arrangement; social support/caregiving; physical and cognitive comorbidities), the contextual level (urban/rural residence; healthcare access; survey year) and the study level such as sampling frame and response rate; setting: community/clinic/institution; measurement instrument and cut-off used, for example, Geriatric Depression Scale (GDS), Patient Health Questionnaire-9 (PHQ-9), Hospital Anxiety and Depression Scale (HADS), Generalized Anxiety Disorder 7-item scale (GAD-7), Depression Anxiety Stress Scales (DASS) and mode of administration; Where available, adjusted estimates will be preferred. We will assess residual confounding using prespecified subgroup analyses and, where data permit, meta-regression (eg, by age structure, sex composition, setting, instrument/cut-off, types of community, country, survey year) will be performed. We will exclude studies at high risk of bias or with non-validated translations or extreme cut-offs to evaluate the robustness of pooled estimates. We will also distinguish confounders from effect modifiers in interpretation.

### Statistical analysis

Pooled prevalence and incidence rate of anxiety, depression and stress will be calculated with 95% confidence intervals CIs. Between-study heterogeneity will be assessed using Cochran’s Q (χ²) and quantified with I² statistics. A random-effects model will be applied to account for variability between studies.^30^ Fixed-effects models will be applied if heterogeneity is minimal. If quantitative synthesis is not feasible due to heterogeneity, a narrative synthesis will be undertaken, summarising the findings descriptively. Subgroup analyses will be conducted to explore variations by: Country or geographical subregion; Study design (cross-sectional, cohort, case–control); participant characteristics (age group, gender, education, socioeconomic status). For estimating pooled prevalence and incidence, we will use point estimates of depression, anxiety and stress along with CI and standard error (SE) from each study. The pooled prevalence and incidence will be estimated across study design, participants’ characteristics and by country. Trend analysis will be performed to estimate change in the burden of depression, anxiety and stress (prevalence/incidence rates) over time (publishing years). Publication bias will be evaluated using funnel plots if 10 or more studies are included in the pooled estimate. Egger’s test will also be applied to statistically assess asymmetry.^31^ Meta-analyses will be performed when ≥2 studies per outcome are sufficiently homogeneous in PECOS and design. Heterogeneity will be explored with prespecified subgroup analyses and, random-effects meta-regression will be conducted when at least ten studies contribute to the outcome.

### Sensitivity analysis

We will assess the robustness of pooled prevalence or incidence of mental disorders with several sensitivity checks. First, we will re-run the random‐effects meta-analysis using alternative proportion models and transformations (Freeman–Tukey double arcsine, logit, untransformed) and a binomial generalised linear mixed model. We will conduct leave-one-out and influence diagnostics, refitting after excluding outliers and studies that materially shifted the pooled estimate. To test design and measurement assumptions, we will restrict analyses to studies at low risk of bias, to probability sampled population surveys and to those using standardised diagnostic interviews Diagnostic and Statistical Manual of Mental Disorders (DSM) / International Classification of Diseases and Related Health Problems(ICD) versus screening instruments or different cut-offs. We will also compare point, 12-month and lifetime prevalence and harmonised case definitions. Potential small-study/publication effects will be examined via funnel plots with regression-based tests and trim-and-fill where appropriate. Results deemed robust if pooled prevalence changed <20% and substantive conclusions across subgroups/time points were unchanged. All statistical analyses will be conducted using software such as The STATA software package (V.14; STATA).

### Ethics and dissemination

As this is a systematic review protocol based on publicly available data from peer-reviewed studies, no primary data collection will be conducted. Therefore, ethical approval is not required for the review itself. Only studies that meet the inclusion criteria and adhere to ethical guidelines in their research design will be included in the review. The findings from the systematic review will be disseminated through peer-review publication and conference presentation.

### Patient and public involvement

Patients and the public were not engaged in the design, execution, reporting or dissemination strategy of this research.

## Discussion

The burden of depression, anxiety and stress among the older adults represents a significant yet underexplored public health concern in the WHO SEARO. This systematic review will provide a comprehensive estimate of the prevalence and incidence of depression, anxiety and stress among the older adult’s population in the region, offering valuable insights into their impact. This protocol outlines the methodology that will be used to synthesise existing evidence, a crucial step in addressing the gaps in research, policy and practice. In this region, a rapidly ageing population, limited healthcare infrastructure and cultural stigmas increase the burden of depression, anxiety and stress among older adults.^32 33^ These factors contribute to the under-reporting of mental health issues, which further complicates the situation.

The available data on the prevalence and incidence of depression, anxiety and stress in the older adult population are fragmented and inconsistent, varying widely across countries and study designs. This review will evaluate each study systematically to estimate the pooled prevalence and incidence of anxiety, depression and stress among older adults.^34^ Through systematic synthesis, this review will compile the findings to produce region-specific estimates that can better inform public health policy making and service planning. The review will explore the variations of the burden of these conditions across different characteristics of older adults and any sociocultural perception for mental health. The JBI tool and GRADE approach will ensure the high quality of evidence and transparency of synthesis. This analysis will help clarify the extent of heterogeneity in the available data. Knowledge gaps will be identified at the region and country level.

The WHO SEARO has two current frameworks that work with mental health for older adults. (1) Regional Mental Health Action Plan 2023–2030, which calls for scaling up community-based services, integrating mental health into primary care, strengthening suicide prevention, improving governance/financing and monitoring across Member States.^35^ Regional Strategy on Healthy Ageing 2024–2030, which aligns with the UN Decade of Healthy Ageing and prioritises age-friendly environments, integrated care and better data to guide action for people in the ‘second half of life’.^36^ This review will directly support policy and planning in the WHO SEARO by pooling region-specific prevalence estimates for mental disorders in older adults. We will provide baseline figures that WHO-SEARO countries can use to track progress towards the 2023–2030 goals set in the Regional Mental Health Action Plan and the Healthy Ageing Strategy. These estimates across country, setting, sex and measurement approach will help identify high-risk groups and service gaps, inform priorities for suicide prevention and guide the integration of screening and care in primary and community services under the Mental Health Gap Action Programme (mhGAP) approach. The findings can be used to set realistic coverage targets, plan workforce and training needs and direct resources to underserved areas. By showing where data are missing or weak, the review also points to improvements needed in routine information systems and monitoring. Together, this evidence may offers practical, actionable input for targeted interventions, capacity-building and evidence-based policies to improve mental health outcomes for older adults across member countries of WHO SERO.

## Supplementary material

10.1136/bmjopen-2025-106812 online supplemental file 1

10.1136/bmjopen-2025-106812 online supplemental file 2
