# Supplementary material for: Burden of anxiety, depression and stress among older adults living in South-East Asia: Protocol for a systematic review and meta-analysis
Source: BMJ Open. 2025 Nov 26;15(11):e106812. doi: 10.1136/bmjopen-2025-106812 (PMC12658544; doi:10.1136/bmjopen-2025-106812)
Supplement: online supplemental file 2 [file bmjopen-15-11-s002.docx]

**Additional file 2:** Search strategies for all databases

**PubMed**

(((((((((((((((((((((((((((Anxiety[MeSH Terms]) OR (Anxiety Disorders[MeSH Terms])) OR (Generalized Anxiety Disorder[MeSH Terms])) OR (Phobia, Social[MeSH Terms])) OR (anxiety[Title/Abstract])) OR (anxious[Title/Abstract])) OR (anxiety disorder*[Title/Abstract])) OR (generalized anxiety disorder[Title/Abstract])) OR (GAD[Title/Abstract])) OR (panic disorder[Title/Abstract])) OR (social anxiety[Title/Abstract])) OR (social phobia[Title/Abstract])) OR (specific phobia[Title/Abstract])) OR (agoraphobia[Title/Abstract])) OR (separation anxiety[Title/Abstract])) OR (test anxiety[Title/Abstract])) OR (trait anxiety[Title/Abstract])) OR (state anxiety[Title/Abstract])) OR (anxiety symptoms[Title/Abstract])) OR (nervousness[Title/Abstract])) OR (apprehension[Title/Abstract])) OR (worry[Title/Abstract])) OR (restlessness[Title/Abstract])) OR (fear[Title/Abstract])) OR (stress[Title/Abstract])) OR (psychological distress[Title/Abstract])) OR (anxiousness[Title/Abstract]))) OR (((((((((((((((((((((((((Stress, Psychological[MeSH Terms]) OR (Stress, Physiological[MeSH Terms])) OR (Stress Disorders, Post‐Traumatic[MeSH Terms])) OR (Stress Disorders, Traumatic[MeSH Terms])) OR (Burnout, Professional[MeSH Terms])) OR (Burnout, Psychological[MeSH Terms])) OR (Psychological Distress[MeSH Terms])) OR (Stress[Title/Abstract])) OR (psychological stress[Title/Abstract])) OR (emotional stress[Title/Abstract])) OR (mental stress[Title/Abstract])) OR (stress response[Title/Abstract])) OR (stress symptoms[Title/Abstract])) OR (chronic stress[Title/Abstract])) OR (acute stress[Title/Abstract])) OR (stressful event*[Title/Abstract])) OR (life stress[Title/Abstract])) OR (perceived stress[Title/Abstract])) OR (stress level*[Title/Abstract])) OR (stress burden[Title/Abstract])) OR (caregiver stress[Title/Abstract])) OR (occupational stress[Title/Abstract])) OR (financial stress[Title/Abstract])) OR (daily hassles[Title/Abstract])) OR (burnout[Title/Abstract]))) AND ((((((((((((((((Aged[MeSH Terms]) OR (Aged, 80 and over[MeSH Terms])) OR (Geriatrics[MeSH Terms])) OR (Frailty[MeSH Terms])) OR (Frail Elderly[MeSH Terms])) OR (older adults[Title/Abstract])) OR (elderly[Title/Abstract])) OR (aging population[Title/Abstract])) OR (senior citizens[Title/Abstract])) OR (aged people[Title/Abstract])) OR (older persons[Title/Abstract])) OR (geriatric patients[Title/Abstract])) OR (aged[Title/Abstract])) OR (older individuals[Title/Abstract])) OR (frail elderly[Title/Abstract])) OR (advanced age[Title/Abstract]))) AND (((((((((((Bangladesh[Title/Abstract]) OR (Bhutan[Title/Abstract])) OR (Democratic People's Republic of Korea[Title/Abstract])) OR (India[Title/Abstract])) OR OR (Maldives[Title/Abstract])) OR (Myanmar[Title/Abstract])) OR (Nepal[Title/Abstract])) OR (Sri Lanka[Title/Abstract])) OR (Thailand[Title/Abstract])) OR (Timor-Leste[Title/Abstract]))

**Web of Science**

1: TS=("Depressive Disorder, Major" OR

"Depressive Disorder" OR

"Dysthymic Disorder" OR

dysthymia OR

"mood disorder*" OR

"affective disorder*" OR

"unipolar depression" OR

"clinical depression" OR

"endogenous depression" OR

melancholia OR

"psychotic depression" OR

"reactive depression" OR

"seasonal depression" OR

"depression symptoms" OR

"depressive episode*" OR

"depressive symptom*" OR

"major depressive disorder" OR

MDD)

2: TS=(anxiety OR

anxious OR

"anxiety disorder*" OR

"generalized anxiety disorder" OR

GAD OR

"panic disorder" OR

"social anxiety" OR

"social phobia" OR

"specific phobia" OR

agoraphobia OR

"separation anxiety" OR

"test anxiety" OR

"trait anxiety" OR

"state anxiety" OR

"anxiety symptoms" OR

nervousness OR

apprehension OR

worry OR

restlessness OR

tension OR

fear OR

stress OR

"psychological distress" OR

anxiousness)

3: TS=(stress OR

"psychological stress" OR

"emotional stress" OR

"mental stress" OR

"stress response" OR

"stress symptoms" OR

"chronic stress" OR

"acute stress" OR

"stressful event*" OR

"life stress" OR

"perceived stress" OR

"stress level*" OR

"stress burden" OR

"caregiver stress" OR

"occupational stress" OR

"financial stress" OR

"daily hassles" OR

burnout)

4: TS=("older adults" OR

elderly OR

"aging population" OR

"senior citizens" OR

"aged people" OR

"older persons" OR

"geriatric patients" OR

aged OR

"older individuals" OR

"frail elderly" OR

"advanced age")

5: TS=(Bangladesh OR

Bhutan OR

"Democratic People's Republic of Korea" OR

India OR

Maldives OR

Myanmar OR

Nepal OR

"Sri Lanka" OR

Thailand OR

"Timor-Leste")

6: #1 OR #2 OR #3

7: #4 AND #5 AND #6

Scopus

**Scopus**

(TITLE-ABS("anxiety") OR

TITLE-ABS("anxious") OR

TITLE-ABS("anxiety disorder*") OR

TITLE-ABS("generalized anxiety disorder") OR

TITLE-ABS("GAD") OR

TITLE-ABS("panic disorder") OR

TITLE-ABS("social anxiety") OR

TITLE-ABS("social phobia") OR

TITLE-ABS("specific phobia") OR

TITLE-ABS("agoraphobia") OR

TITLE-ABS("separation anxiety") OR

TITLE-ABS("test anxiety") OR

TITLE-ABS("trait anxiety") OR

TITLE-ABS("state anxiety") OR

TITLE-ABS("anxiety symptoms") OR

TITLE-ABS("nervousness") OR

TITLE-ABS("apprehension") OR

TITLE-ABS("worry") OR

TITLE-ABS("restlessness") OR

TITLE-ABS("fear") OR

TITLE-ABS("stress") OR

TITLE-ABS("psychological distress") OR

TITLE-ABS("anxiousness") OR

TITLE-ABS("psychological stress") OR

TITLE-ABS("emotional stress") OR

TITLE-ABS("mental stress") OR

TITLE-ABS("stress response") OR

TITLE-ABS("stress symptoms") OR

TITLE-ABS("chronic stress") OR

TITLE-ABS("acute stress") OR

TITLE-ABS("stressful event*") OR

TITLE-ABS("life stress") OR

TITLE-ABS("perceived stress") OR

TITLE-ABS("stress level*") OR

TITLE-ABS("stress burden") OR

TITLE-ABS("caregiver stress") OR

TITLE-ABS("occupational stress") OR

TITLE-ABS("financial stress") OR

TITLE-ABS("daily hassles") OR

TITLE-ABS("burnout")

)

AND

(TITLE-ABS("older adults") OR

TITLE-ABS("elderly") OR

TITLE-ABS("aging population") OR

TITLE-ABS("senior citizens") OR

TITLE-ABS("aged people") OR

TITLE-ABS("older persons") OR

TITLE-ABS("geriatric patients") OR

TITLE-ABS("aged") OR

TITLE-ABS("older individuals") OR

TITLE-ABS("frail elderly") OR

TITLE-ABS("advanced age")

)

AND

(TITLE-ABS("Bangladesh") OR

TITLE-ABS("Bhutan") OR

TITLE-ABS("Democratic People's Republic of Korea") OR

TITLE-ABS("India") OR

TITLE-ABS("Maldives") OR

TITLE-ABS("Myanmar") OR

TITLE-ABS("Nepal") OR

TITLE-ABS("Sri Lanka") OR

TITLE-ABS("Thailand") OR

TITLE-ABS("Timor-Leste"))

PsyINFO

1. , Major Depressive Disorder.mp. or Depressive Disorder/ or Dysthymic Disorder.mp. or dysthymia.mp. or mood disorder*.mp. or affective disorder*.mp. or unipolar depression.mp. or clinical depression.mp. or endogenous depression.mp. or melancholia.mp. or psychotic depression.mp. or reactive depression.mp. or seasonal depression.mp. or depression symptoms.mp. or depressive episode.mp. [mp=title, abstract, heading word, table of contents, key concepts, original title, tests & measures, mesh word]
2. (anxiety or anxious or anxiety disorder* or generalized anxiety disorder or GAD or panic disorder or social anxiety or social phobia or specific phobia or agoraphobia or separation anxiety or test anxiety or trait anxiety or state anxiety or anxiety symptoms or nervousness or apprehension or worry or restlessness or tension or fear or stress or psychological distress or anxiousness).mp. [mp=title, abstract, heading word, table of contents, key concepts, original title, tests & measures, mesh word]
3. (Stress or psychological stress or emotional stress or mental stress or stress response or stress symptoms or chronic stress or acute stress or stressful event* or life stress or perceived stress or stress level* or stress burden or caregiver stress or occupational stress or financial stress or daily hassles or burnout).mp. [mp=title, abstract, heading word, table of contents, key concepts, original title, tests & measures, mesh word]
4. (Aged or older adults or elderly or aging population or senior citizens or aged people or older persons or geriatric patients or aged or older individuals or frail elderly or advanced age or Geriatrics or Frailty).mp. [mp=title, abstract, heading word, table of contents, key concepts, original title, tests & measures, mesh word]
5. (Bangladesh or Bhutan or Democratic People's Republic of Korea or India or Maldives or Myanmar or Nepal or Sri Lanka or Thailand or Timor-Leste).mp. [mp=title, abstract, heading word, table of contents, key concepts, original title, tests & measures, mesh word] 43065
6. 1 or 2 or 3
7. 4 and 5 and 6

**Additional file 3: The below checklist will be utilized for the quality assessment of the articles.**

JBI critical appraisal checklist for observational studies

| Items | Yes | No | Unclear | Not applicable | Support for judgement |
| --- | --- | --- | --- | --- | --- |
| 1. Were the criteria for inclusion in the sample clearly defined? | □ | □ | □ | □ |  |
| 1. Were the study subjects and the setting described in detail? | □ | □ | □ | □ |  |
| 1. Was the exposure measured in a valid and reliable way? | □ | □ | □ | □ |  |
| 1. Were objective, standard criteria used for measurement of the condition? | □ | □ | □ | □ |  |
| 1. Were confounding factors identified? | □ | □ | □ | □ |  |
| 1. Were strategies to deal with confounding factors stated? | □ | □ | □ | □ |  |
| 1. Were the outcomes measured in a valid and reliable way? | □ | □ | □ | □ |  |
| 1. Was appropriate statistical analysis used? | □ | □ | □ | □ |  |
