# Supplementary material for: Burden of anxiety, depression and stress among older adults living in South-East Asia: Protocol for a systematic review and meta-analysis
Source: BMJ Open. 2025 Nov 26;15(11):e106812. doi: 10.1136/bmjopen-2025-106812 (PMC12658544; doi:10.1136/bmjopen-2025-106812)
Supplement: Supplementary file 3 [file bmjopen-2025-106812.reviewer_comments.pdf]

PEER REVIEW HISTORY

BMJ Open publishes all reviews undertaken for accepted manuscripts. Reviewers are asked to complete a checklist review form and are provided with free text boxes to elaborate on their assessment. These free text comments are reproduced below.

ARTICLE DETAILS

Title (Provisional)

The burden of anxiety, depression and stress among the older adults living in South-East Asia: A protocol for systematic review and meta-analysis

Authors

ISLAM, MD SAIMUL; Patel, Jeetesh V; Ahmed, Helal Uddin; Naheed, Aliya; Gill, Paramjit

---

VERSION 1 - REVIEW

**Reviewer**	**1**
**Name**	**Saha , Indranil**
**Affiliation**	**Indian Council of Medical Research**
**Date**	**19-Aug-2025**
**COI**	**None**

The manuscript needs the following clarifications:

1. How is it decided that the CMD consists of only anxiety, depression and stress among older adults?

2. Better to replace the term ‘elderly’ by ‘older adults’

3. Statistical analysis: It seems to have ended with meta-analysis, but in the title, it is mentioned as a systematic review only. Pooled estimate, random model, etc. is mentioned – please clarify / correct

4. In one area, it is written that the search will be conducted up to May 2024 (abstract), but in another area, it is mentioned as May 2025. – Please make it uniform.

5. What are the countries included under the WHO South East Asia? Please mention.

6. Why will existing systematic review studies be included in the present systematic review? – Usually, it is not taken.

7. Mention the current policy/recommendations available for Mental health among older adults in South East Asian regions, and how this SRMA will contribute to that.

8. It is unclear whether a sensitivity analysis will be performed or not?

9. Please mention about potential confounding variables that might influence the findings.

10. Include one relevant reference for the present manuscript – DOI: 10.4103/jncd.jncd_34_24 (Das D et al).

11. Is there any time frame for the search strategy?

12. Search strategy – basically there would be no intervention or exposure as mentioned. It will be listed in the outcome only. Thus, in PICOS, there will be Population, Outcome and Settings (SEA region) – please correct.

VERSION 1 - AUTHOR RESPONSE

Reviewer: 1

The manuscript needs the following clarifications:
1. How is it decided that the CMD consists of only anxiety, depression and stress among older adults?

Response: We acknowledge that there are many mental disorders commonly reported across different population groups. In this study we operationalized "common mental disorders" as symptoms of depression, anxiety, and stress grounded in public health usage and measurement. First, the World Health Organization identifies anxiety and depressive disorders as the most prevalent mental disorders globally (1). Second, ICD-11 includes a dedicated grouping "Disorders specifically associated with stress" indicating that stress-related psychopathology is an established diagnostic domain (2). Third, these conditions are **highly prevalent in older adults and contribute to global burden of diseases, but not explored in the Southeast Asia region (3).** We have updated the manuscript to explicitly include depression, anxiety, and stress where relevant, in both the title and main text.

References

1. WHO. Mental disorders 2022 [Available from: https://www.who.int/news-room/fact-sheets/detail/mental-disorders#:~:text=In%202019%2C%201%20in%20every,and%20violations%20of%20human%20rights.

2. Maercker A, Eberle DJ. Disorders specifically associated with stress in ICD-11. Clinical psychology in Europe. 2022;4(Spec Issue):e9711.

3. Uphoff EP, Newbould L, Walker I, Ashraf N, Chaturvedi S, Kandasamy A, et al. A systematic review and meta-analysis of the prevalence of common mental disorders in people with non-communicable diseases in Bangladesh, India, and Pakistan. J Glob Health. 2019;9(2):020417.

2. Better to replace the term 'elderly' by 'older adults'

Response: Thank you for this suggestion and done.

3. Statistical analysis: It seems to have ended with meta-analysis, but in the title, it is mentioned as a systematic review only. Pooled estimate, random model, etc. is mentioned – please clarify / correct

Response: Thank you we have amended the title as:

The burden of anxiety, depression and stress among the older adults living in South-East Asia: A protocol for systematic review and meta-analysis

4. In one area, it is written that the search will be conducted up to May 2024 (abstract), but in another area, it is mentioned as May 2025. – Please make it uniform.

Response: Thank you and this has been amended. (Page 1 & 6)

5. What are the countries included under the WHO South East Asia? Please mention.

Response: Thank you for this:

"The WHO South-East Asia Region comprises the following Member States: Bangladesh, Bhutan, Democratic People's Republic of Korea, India, Maldives, Myanmar, Nepal, Sri Lanka, Thailand, and Timor-Leste." (Page 4, 6 & 7)

6. Why will existing systematic review studies be included in the present systematic review? – Usually, it is not taken.

Response: We agree with the reviewers' observation. In this study, we will include primary observational studies for quantitative evidence synthesis. Existing systematic reviews will be used only to enhance retrieval of primary studies (backward and forward citation searching) and to contextualize our findings. They will not be included as studies in the meta-analysis or narrative synthesis. Now we have clarified in the revised version as below.
"We will include peer-reviewed primary observational studies including longitudinal cohort, cross-sectional, case–control studies that report depression, anxiety, stress outcomes in older adults. We will screen existing systematic reviews of prevalence or incidence only to identify additional eligible primary studies and to contextualize our findings; these reviews will be documented as background sources and not included in the evidence synthesis." (Page 7)

7. Mention the current policy/recommendations available for Mental health among older adults in South East Asian regions, and how this SRMA will contribute to that.

Response: We appreciate the reviewer suggestion about policy and recommendation. Now we have added a paragraph about the recommendation of policy and how this study will contribute. The added paragraph under discussion section reads as below.

"The WHO SEARO has two current frameworks that works with mental health for older adults. (1) Regional Mental Health Action Plan 2023–2030, which calls for scaling up community-based services, integrating mental health into primary care, suicide prevention, strengthened governance/financing, and monitoring across Member States (35). Regional Strategy on

Healthy Ageing 2024–2030, which aligns with the UN Decade of Healthy Ageing and prioritizes age-friendly environments, integrated care, and better data to guide action for people in the "second half of life"(36). This review will directly support policy and planning in the WHO South-East Asia Region (SEARO). By pooling region-specific prevalence estimates for mental disorders in older adults. We will provide baseline figures that WHO-SEARO countries can use to track progress toward the 2023–2030 goals set in the Regional Mental Health Action Plan and the Healthy Ageing Strategy. These estimates across country, setting, sex, and measurement approach will help identify high risk groups and service gaps, inform priorities for suicide prevention, and guide the integration of screening and care in primary and community services under the mhGAP approach. The findings can be used to set realistic coverage targets, plan workforce and training needs, and direct resources to underserved areas. By showing where data are missing or weak, the review also points to improvements needed in routine information systems and monitoring. Together, this evidence offers practical, actionable input for targeted interventions, capacity-building, and evidence-based policies to improve mental health outcomes for older adults across member countries of WHO South East Asian Region." (Page 12)

Reference

35. WHO-SEARO. Mental health action plan for the WHO South-East Asia Region 2023–2030. 2023.
36. WHO-SEARO. WHO South-East Asia Regional Strategy on Healthy Ageing 2024–2030. 2023.

8. It is unclear whether a sensitivity analysis will be performed or not?

Response: Thank you for this and the new paragraph reads:

"Sensitivity analysis: We will assess the robustness of pooled prevalence or incidence of mental disorders with several sensitivity checks. First, we will re-run the random-effects meta-analysis using alternative proportion models and transformations (Freeman–Tukey double arcsine, logit, untransformed) and a binomial generalized linear mixed model. We will conduct leave-one-out and influence diagnostics, refitting after excluding outliers and studies that materially shifted the pooled estimate. To test design and measurement assumptions, we will restrict analyses to studies at low risk of bias, to probability sampled population surveys and to those using standardized diagnostic interviews (DSM/ICD) versus screening instruments or different cut-offs. We will also compare point, 12-month, and lifetime prevalence and harmonized case definitions. Potential small-study/publication effects will be examined via funnel plots with regression-based tests and trim-and-fill where appropriate. We considered results robust if the pooled prevalence changed by <20% and the main conclusions across subgroups or time points stayed the same." (Page 10 & 11)

9. Please mention about potential confounding variables that might influence the findings.

Response: This now reads as:

Potential confounding variables: We will extract potential confounders at the individual level (age bands within older adults; sex; socioeconomic status; marital/living arrangement; social support/caregiving; physical and cognitive comorbidities;), the contextual level (urban/rural

residence; health-care access; survey yea, and the study level such as sampling frame and response rate; setting: community/clinic/institution; measurement instrument and cut-off used e.g., GDS, PHQ-9, HADS, GAD-7, DASS-21 and mode of administration; Where available, adjusted estimates will be preferred. We will assess residual confounding using pre-specified subgroup analyses and, where data permit, meta-regression (e.g., by age structure, sex composition, setting, instrument/cut-off, types of community, country, survey year) will be performed. We will exclude studies at high risk of bias or with non-validated translations or extreme cut-offs to evaluate the robustness of pooled estimates. We will also distinguish confounders from effect modifiers in interpretation." (Page 9)

10. Include one relevant reference for the present manuscript – DOI: 10.4103/jncd.jncd_34_24 (Das D et al).

Response: We thank the reviewer for this citation which has been added in page 4

11. Is there any time frame for the search strategy?

Response: Thank you and this has been addressed above in response to the Editor.

12. Search strategy – basically there would be no intervention or exposure as mentioned. It will be listed in the outcome only. Thus, in PICOS, there will be Population, Outcome and Settings (SEA region) – please correct.

Response: We appreciate the suggestion of the reviewer. We have defined eligibility criteria based on PECOS which includes population, exposure, comparator, outcome and settings. We have added a table to define this criterion. We have added a separate table for PECOS in page 5:

Table 1. Eligibility criteria for including studies

PECOS elements	*Criteria*
Population	Individuals aged ≥60 years
Exposure	Presence of anxiety, depression or stress
Comparator	Not applicable
Outcome	Prevalence /incidence; associated factors
Settings	Member countries of WHO-SEARO region: Bangladesh, Bhutan, Democratic People's Republic of Korea, India, Maldives, Myanmar, Nepal, Sri Lanka, Thailand, and Timor-Leste

VERSION 2 - REVIEW

**Reviewer**	**1**
**Name**	**Saha , Indranil**
**Affiliation**	**Indian Council of Medical Research**
**Date**	**13-Oct-2025**

COI

The authors have satisfactorally addresed all the previous queries. Only one question: Population is written as older adults aged 60 years or above. Whether all the older adults i.e. community dwelling / OPD based / IPD based hospital / suffereing from oher diseases will be taken into consideration?

VERSION 2 - AUTHOR RESPONSE

- The authors have satisfactorally addresed all the previous queries. Only one question: Population is written as older adults aged 60 years or above. Whether all the older adults i.e. community dwelling / OPD based / IPD based hospital / suffereing from oher diseases will be taken into consideration?.

Response: We thank the reviewer for this helpful clarification. We agree with the concern and confirm that all individuals aged 60 years and older will be included, specifically community-dwelling adults, those receiving outpatient or inpatient hospital care, and individuals with or without comorbid conditions. We have revised the methodology accordingly. The updated text reads:

"population: individuals aged 60 years and older from countries in the WHO South-East Asia Region, including community-dwelling older adults and those attending outpatient clinics or admitted to hospitals, with or without other health conditions" (Page 5)
